# Additional impact of concomitant hypertension and osteoarthritis on quality of life among patients with type 2 diabetes in primary care in Germany – a cross-sectional survey

**DOI:** 10.1186/1477-7525-7-19

**Published:** 2009-02-27

**Authors:** Antje Miksch, Katja Hermann, Andreas Rölz, Stefanie Joos, Joachim Szecsenyi, Dominik Ose, Thomas Rosemann

**Affiliations:** 1 Department of general practice and health services research, University Hospital of Heidelberg, Heidelberg, Germany

## Abstract

**Background:**

Patients with type 2 diabetes are likely to have comorbid conditions which represent a high burden for patients and a challenge for primary care physicians. The aim of this cross-sectional survey was to assess the impact of additional comorbidities on quality of life within a large sample of patients with type 2 diabetes in primary care.

**Methods:**

A cross-sectional survey within a large sample (3.546) of patients with type 2 diabetes in primary care was conducted. Quality of life (QoL) was assessed by means of the Medical Outcome Study Short Form (SF-36), self reported presence of comorbid conditions was assessed and groups with single comorbidities were selected. QoL subscales of these groups were compared to diabetes patients with no comorbidities. Group comparisons were made by ANCOVA adjusting for sociodemographic covariates and the presence of depressive disorder.

**Results:**

Of 3546 questionnaires, 1532 were returned, thereof 1399 could be analysed. The mean number of comorbid conditions was 2.1. 235 patients declared to have only hypertension as comorbid condition, 97 patients declared to have osteoarthritis only. Patients suffering from diabetes and hypertension reached similar scores like diabetic patients with no comorbidities. Patients with diabetes and osteoarthritis reached remarkable lower scores in all subscales. Compared to patients with diabetes alone these differences were statistically significant in the subscales representing pain and physical impairment.

**Conclusion:**

The impact of osteoarthritis as an often disabling and painful condition on QoL in patients with type 2 diabetes is higher than the impact of hypertension as common but often asymptomatic comorbidity. Individual care of patients with chronic conditions should aim at both improving QoL and controlling risk factors for severe complications.

## Introduction

Diabetes represents one of the major challenges for health care systems all over the world while consuming a lot of health care resources. Furthermore, some estimates predict a global increase in the number of patients suffering from diabetes from 135 to 300 million patients until the year 2025 [1]. Most diabetes patients suffer from type 2 diabetes.

Quality of life (QoL) in patients with diabetes is reduced and patients are impaired in nearly all domains of daily life [2,3]. In addition patients with diabetes are more likely to suffer from comorbid conditions such as hypertension, myocardial infarction or stroke as persons without diabetes [4]. Little is known about the additional impact of comorbid conditions on QoL in diabetics, especially in unselected patients as in primary care [5,6]. With increasing age QoL depends more and more on the individual health status and resulting impairments [7-9]. In general practice it is "the rule rather than the exception" to see patients with more than a single chronic condition [10]. The high prevalence of multimorbidity constitutes a high burden for the patients and a challenge for primary care physicians simultaneously. As a consequence it is often difficult to attribute impairments in health related quality of life to one particular disease or chronic condition [11,12].

The aim of this cross-sectional survey was to assess quality of life by means of the Medical Outcome Study Short Form (SF-36) with regard to differences in the additional impact of common comorbidities within a large sample of patients with type 2 diabetes in primary care. In order to assess the possible impact of particular conditions patient groups with single comorbidities were selected.

## Methods

This cross-sectional survey among patients with type 2 diabetes has been conducted as part of the ELSID study (Evaluation of a Large Scale Implementation of Disease Management Programmes for patients with type 2 diabetes) [13]. Study protocols of the ELSID-study and the presented survey were both approved by the ethics committee of the University of Heidelberg.

### Participants

Based on the total sample observed in the ELSID-study (n = 20.625, 59,2% female) a random sample of 3546 patients (59,3% female) was drawn. All participants were patients with type 2 diabetes and insured by one large statutory regional health care fund called Allgemeine Ortskrankenkasse (AOK) which covers about 40% of the German population. The criteria for including patients in the ELSID study are described elsewhere [13]. For the purpose of this survey patients were addressed directly by their health insurance in November 2006 and received the questionnaire and a postage-paid envelope addressed to the study center. In order to ensure a high level of data privacy patients were asked to return the completed questionnaires which were only labelled with a unique pseudonym for each patient directly to the University of Heidelberg. Patients were informed that returning the questionnaire would be assumed as consent for scientific analysis of the answers. They were informed that neither their GP nor the health insurance could get knowledge about individual answers. Two weeks later, all patients received a reminder (without questionnaire) regardless if they had sent their questionnaire back or not. All patients could participate in the draw of a prize of 6 times EURO 250 (approximately USD 375) by sending in a separate postage-paid return envelope to the study centre. This procedure was completely separated from the questionnaires in order to assure confidentiality.

Based on sociodemographics out of routine claims data of the statutory health insurance we performed a non-responder analysis including age and gender of all addressed patients. Identification for this comparison was based on the unique pseudonym.

### Data collection

The questionnaire included the German versions of the Medical Outcome Study Short Form (SF-36) and the 9-item Patient Health Questionnaire (PHQ-9) as well as sociodemographic questions.

The SF-36 is a generic questionnaire for measuring health-related QoL, which is often used in international studies. [14,15] The SF-36 provides scores in eight domains (Physical functioning (PF), Role-physical (RP), Bodily Pain (BP), General Health (GH), Vitality (VT), Social Functioning (SF), Role-Emotional (RE) and Mental Health (ME)).

In addition two summary measures labelled as the Physical component summary scale (PCS) and the Mental component summary scale (MCS) [14,15] can be calculated. The scores range from 0 to 100, higher values represent a better QoL. We compared the results of the present sample of patients with type 2 diabetes with data of the general population extracted out of the German National Health Interview and Examination Survey [16]. Therefore, according to normative data we divided the study sample into 4 age groups (50–59, 60–69, 70–79, 80 and more).

The 9-item Patient Health Questionnaire (PHQ-9) is a self-administered, well validated and widely used diagnostic instrument to assess depressive symptoms and severity of depressive disorders [17,18]. It provides a summary score ranging from 0 to 27, with higher values indicating higher severity. A cut-off value of 10 has been reported to have a sensitivity of 0.88 and a specificity of 0.88 [18].

Sociodemographic data included age, gender, educational level, occupational status, partnership/marital status and the monthly household-income. Furthermore, self-administered information about the presence of the following conditions was collected: hypertension, coronary heart disease, myocardial infarction, congestive heart failure, stroke, asthma, chronic bronchitis, gastric ulcer, cancer and osteoarthritis. Out of this information we calculated the mean total number of conditions and selected patient groups with the most frequently declared single comorbidities.

In order to calculate the body mass index (BMI) we recorded height and weight of the patients. We assessed the socioeconomic status (SES) with a non-weighted social class index based on the three dimensions education, occupation and household-income. Based on a score with possible ranges from 3 to 21 points three social classes (lower, middle, upper) were defined [19].

### Statistical analysis

All statistical analyses were performed using the SPSS software program (version 15.0). Unadjusted group comparisons of continuous variables (reported in terms of means and standard deviations) were made using the student's t test or the Mann-Whitney-Test as appropriate. Normality of distribution was tested by means of the Kolmogorov-Smirnov test. The chi-square test was used for categorial variables. For the analysis of an additional impact of specific comorbid conditions on QoL we selected patient groups with one single comorbid condition. Differences between these groups were analysed by ANCOVA adjusting for possible confounders that may have an influence. These covariates were age (50–59 years, 60–69 years, 70–79 years, > 80 years), gender, SES (lower, middle, upper social class), BMI (<25, 25–30, >30) and depressive disorder (<10, ≥ 10) . To avoid effects of multiple testing post hoc corrections according to Bonferroni were performed. The level of significance was defined as p < 0.05.

## Results

1532 of 3546 questionnaires were returned (response rate 43.2%), 1399 were eligible for further analysis.

### Non-Responder-analysis

Responder were younger than non-responder (responder: 70.3 years [95% CI 69.9; 70.7], non-responder 71.8 years [71.4; 72.2]), p < 0.001. Of the responder 686 were male (46.6%) and 787 were female (53.4%); among the non-responder 736 were male (35.5%) and 1337 (64.6%) were female.

### Sociodemographic data

Table 1 shows sociodemographic characteristics of the study sample. Of 1399 included patients 649 were male (46.4%) and 750 were female (53.6%). The mean number of comorbid conditions was 2.1 (range 0–8). 904 patients (64.6%) were married or lived in partnership respectively. 1068 patients (76.3%) were grouped as "low socioeconomic status", according to the mentioned scoring. The number of smokers was 117 (8.4%).

**Table 1:** Sociodemographic characteristics of the study sample

	**Total N = 1399**
**Gender female**	
Number (%)	750 (53.6)
**Age**	
Mean [95% CI]	70.3 (8.5)
**Married/living in partnership**	
No (%)	904 (64.6)
**Socioeconomic Status**	
No. (%)	
Low	1068 (76.3)
Middle	221 (15.8)
High	20 (1.4)
**≤9 years of education**	
Number (%)	998 (71.3)
**Annual income**	
Number (%)	
< 15000	689 (49.2)
15000–36000	632 (45.2)
>36000	78 (5.6)
**Smoker**	
Number (%)	117 (8.4)
**BMI**	
Mean (SD)	30.3 (6.1)
**No. of comorbid conditions**	
Mean (SD)	2.1 (1.4)

SD = Standard deviation

### Health related quality of life

Table 2 shows means for the eight domains of the SF-36 scales and the two component scales for the total sample of patients with type 2 diabetes in comparison to normative data. All data for each of the eight SF-36 subscales were not normally distributed. Compared to the general population QoL was worse in all domains reaching statistical significance in all subscales.

**Table 2:** SF36 scales compared to normative data

		PF	RP	BP	GH	VT	SF	RE	MH	PCS	MCS
Total sample	Total sample N = 1399	51.04 (30.38)	44.50 (44.98)	50.10 (28.91)	47.41 (18.87)	45.23 (21.71)	70.30 (27.26)	63.69 (45.59)	63.84 (21.64)	36.49 (11.65)	47.67 (11.53)
	Norm	85.71 (22.10)	83.70 (31.73)	79.08 (27.38)	68.05 (20.15)	63.27 (18.47)	88.76 (18.40)	90.35 (25.62)	73.88 (16.38)	50.21 (10.24)	51.54 (8.14)
	p-Wert*	<0.001	<0.001	<0.001	<0.001	<0.001	<0.001	<0.001	<0.001	<0.001	<0.001

* p-values in the table concern the comparison to normative data

PF = Physical functioning, RP = Role physical, BP = Bodily pain, GH = General health, VT = Vitality, SF = Social functioning, RE = Role emotional, ME = Mental health, PCS = Physical component scale, MCS = Mental component scale

### Number of Comorbidities

Hypertension (71.6%) and osteoarthritis (57.0%) were the most common comorbid conditions. With declining frequency other conditions were stated as following: coronary vessel disease (20.7%), congestive heart failure (17.3%), chronic bronchitis (10.3%), cancer (8.1%), myocardial infarction (7.5%) stroke (6.2%), asthma (4.3%), gastric ulcer (3.5%).

**Figure 1:**
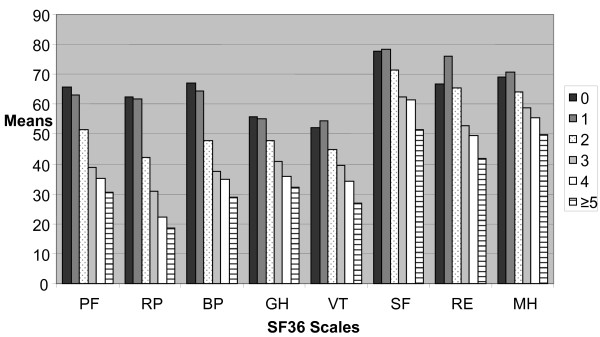
**SF36 subscales depending on the number of comorbidities**. PF = Physical functioning, RP = Role physical, BP = Bodily pain, GH = General health, VT = Vitality, SF = Social functioning, RE = Role emotional, ME = Mental health.

With an increasing number of comorbid conditions, SF36 scales reached lower values as we displayed in figure 1.

### Additional impact of comorbid conditions

Table 3 presents the scores for the SF-36 subscales and the two component scales for diabetics without any comorbid condition as well as for patients with hypertension or osteoarthritis. 147 patients indicated to have only diabetes (mean age 70.3 years [95% CI: 68.80; 71.81], 53.7% female). 235 patients declared to have hypertension as only comorbid condition (mean age 68.02 years [95% CI: 66.94;69.09], 56.2% female). As can be seen patients with hypertension achieve higher scores than patients with diabetes only. Adjusted for age, BMI, gender, SES and depressive disorder these differences did not reach statistical significance neither in the 8 subscales nor in the two component scales. 97 patients declare to have osteoarthritis as only comorbid condition (mean age 69.93 years [95% CI: 68.10; 71.76], 48.5% female). Patients with osteoarthritis had remarkable lower scores in all SF36 domains. Compared to the diabetes patients without comorbidities, the differences were statistically significant in the subscales Physical functioning (p < 0.001), Role physical (p < 0.05), Bodily pain (p < 0.001), General health (p < 0.05), Social functioning (p < 0.05) and furthermore the Physical component scale (p < 0.001). Finally, table 3 displays the scores of 271 patients with both osteoarthritis and hypertension (mean age 69.65 years, [95% CI 68.72; 70.57], 59.0% female), which were similar or higher than those of patients with osteoarthritis alone. Compared to patients without comorbidities all scores were lower reaching statistical significance in Physical functioning (p < 0.001), Role physical (p < 0.05), Bodily pain (p < 0.001), General health (p < 0.01), Vitality (p < 0.05) and the Physical component scale (p < 0.001).

**Table 3:** SF-36 subscales and component scales in patients with diabetes, hypertension and osteoarthritis (all data were mean and SD)

	PF	RP	BP	GH	VT	SF	RE	MH	PCS	MCS
**Diabetes without comorbidity** (n = 147)	65.77 (30.44)	62.42 (44.20)	66.94 (30.26)	55.82 (20.17)	52.09 (23.78)	77.69 (23.82)	66.83 (43.91)	69.21 (21.25)	43.45 (11.38)	48.75 (10.93)
**Diabetes and Hypertension** (n = 235)	70.02 (26.14)	72.21 (40.26)	72.89 (27.01)	57.79 (17.15)	58.33 (21.05)	81.47 (22.52)	82.52 (35.08)	72.79 (17.88)	45.51 (9.52)	51.49 (9.09)
**Diabetes and osteoarthritis** (n = 97)	49.99 *** (27.90)	41.46* (44.56)	44.21*** (21.54)	50.46* (17.06)	47.98 (18.84)	71.60* (26.99)	62.45 (44.46)	65.68 (18.33)	35.30*** (10.50)	48.31 (10.11)
**Diabetes, hypertension and osteoarthritis** (n = 271)	53.08 *** (28.04)	45.50* (45.12)	44.60*** (23.99)	49.13** (18.02)	46.93* (19.42)	74.25 (26.78)	68.06 (44.92)	66.35 (20.83)	35.93*** (11.07)	49.31 (11.80)

PF = Physical functioning, RP = Role physical, BP = Bodily pain, GH = General health, VT = Vitality, SF = Social functioning, RE = Role emotional, ME = Mental health, PCS = Physical component scale, MCS = Mental component scale

All group comparisons are versus Diabetes without comorbidity (adjusted for age, bmi, gender, ses and depressive disorder)

* p < 0.05; ** p < 0.01; *** p < 0.001

## Discussion

In this cross-sectional survey performed in a primary care setting, QoL in patients with type 2 diabetes is significantly lower compared to the general population. Additionally, this study revealed declining scores for all SF-36 subscales with an increasing number of comorbid conditions. The most common comorbid conditions reported were hypertension and osteoarthritis with osteoarthritis having remarkable more impact on quality of life than hypertension.

Over the last two decades health related quality of life, individual health status or well-being have gained more importance as patient-relevant outcome parameters within medical and health services research [7]. Especially for patients suffering from one or several chronic conditions care should focus on the best possible management of the disease and additional impairments on daily life instead of recovery and health. [2,20]. For older patients improvements within QoL may often have a more important role than a possible extension of life time ("add life to years, not years to life") [21,22].

Comparable to results of other studies [3,23-25] patients with type 2 diabetes in our sample were limited in all scores of the SF-36 compared to people without diabetes. According to the literature the number of comorbid conditions was associated with a lower quality of life in all domains of the SF-36 [26,27]. Interestingly in our study patients with hypertension and diabetes achieved higher scores than patients with only diabetes. However, these differences did not reach statistical significance after adjusting for relevant variables. These findings are in accordance with previous studies, describing similar quality of life scales of patients with hypertension and those without any chronic condition [28,29]. One reason for this finding may be that hypertension is often asymptomatic and physically less impairing than other diseases. However, other studies showed hypertensive patients to have lower scales in QoL than normotensive patients because of adverse effects of drugs used in the treatment of the high blood pressure [30] or because of a so called labelling effect [31]. Wee et al. assumed that there are chronic conditions with non-additional effects on health related QoL, so that having both conditions is not more disabling than having one of them [6]. Sprangers et al. describe a mechanism of accommodation to a chronic illness with changes in internal standards and values – the so called "response shift" [12].

It is important to keep in mind that hypertension perhaps does not intensify the burden for the patients since high blood pressure levels represent a major risk factor for cardiovascular mortality and morbidity especially for patients with type 2 diabetes [32]. This has to be taken into account as an additional and important risk factor, both from patients and from physicians [28].

Regarding osteoarthritis as comorbidity we found remarkable lower scales in all domains of the SF-36 in particular within the subscales related to physical well-being. The revealed high burden of patients with osteoarthritis is in accordance with other studies and congruent with the clinical experience of primary care physicians [33-36]. Major problems for patients with osteoarthritis are pain and disability. These symptoms are associated with an increased health service utilization [35,37,38] and have to be kept in mind when dealing with diabetic patients with concomitant osteoarthritis.

The list of self reported comorbidities used in this survey did not contain any mental conditions like e.g. depression, so we were not able to assess the possible impact of these potential comorbidities as we did with somatic comorbidities. However, the used set of questionnaires contained the PHQ-9 as a screening instrument for depressive disorder. This enabled us to control our data for this important issue [12,26]. To evaluate the impact of mental comorbidity on QoL in primary care further research is still needed.

The present study has some limitations. First of all the results were cross-sectional, any conclusions on causality are impossible. All data were self reported, some chronic conditions could be under- or overreported. All questions were filled out self-dependent, considering the mean age of the participants misconceptions could not be excluded. Furthermore calculating the BMI out of self reported height and weight is associated with a limited validity especially in older adults [39,40]. Smoking rates in our sample were self reported too. But there is some evidence that the validity of self-reported smoking within survey studies is reasonable [41]. Furthermore the BMI and the percentage of smokers in our study sample were comparable to findings in the primary care population in the US and Germany [42-44].

The most important limitation might be that we had no knowledge about the severity of the addressed comorbidities. A fact which might limit generalizability of our findings is that all participants of our survey were from the same regional health fund. This insurance fund covers a sample with a higher proportion of elder insurants and a higher prevalence of multimorbidity than other insurers in Germany.

The response rate of our survey was moderate, but a non-responder analysis could be performed, showing that non-responder were slightly older and more likely to be female. The response rates might have been higher if the questionnaires would have been sent out by the university department directly [45] instead of the health insurance fund. However, due to a strict protection of data privacy we weren't able to contact the patients directly.

Strengths of our study were the large and heterogeneous study sample collected in a primary care setting. Since patients' selection was primarily conducted by using routine claims data and secondarily by drawing a random sample selection bias is unlikely.

## Conclusion

This large survey provided a more differentiated view on QoL of patients with type 2 diabetes in primary care regarding the common comorbid conditions hypertension and osteoarthritis and therefore contributes to a better understanding of diabetic patients. The study emphasized that osteoarthritis as a common, disabling and painful comorbid condition has a stronger impact on QoL than hypertension. Individualized care of patients with chronic conditions should consider both improving QoL and controlling risk for severe complications. For primary care physicians this constitutes a challenge with different faces and requires awareness of the patients' differentiated perception. In order to affect QoL in primary care osteoarthritis should get more attention as associated pain and disability are more important from a patients' point of view as hypertension. Simultaneously efforts for advising and patient education should focus on hypertension as asymptomatic but important risk factor. Chronic conditions and multimorbidity are an important and increasing challenge for GPs. So far most studies focussed on the impact of one condition on QoL. As our results suggest it is important to assess several conditions and their impact on individual QoL. This should be considered within further research.

## Competing interests

The authors declare that they have no competing interests.

## Authors' contributions

AM designed and conducted the study and drafted the manuscript. AR performed the data management, AR and KH contributed to the statistical analysis. SJ, JS and TR participated in the study design. KH, SJ, JS and TR contributed substantially to the manuscript. All authors read an approved the final manuscript.
